# ITPKA suppresses glioma progression and predicts patient prognosis

**DOI:** 10.3389/fonc.2026.1802857

**Published:** 2026-04-22

**Authors:** Naiyue Zhang, Min Peng, Jun Liu, Shizhang Ling

**Affiliations:** 1 Department of Neurosurgery, the Second Affiliated Hospital of Wannan Medical College, Wuhu, China; 2 The Translational Research Institute for Neurological Disorders, Department of Neurosurgery, The First Affiliated Hospital of Wannan Medical College (Yijishan Hospital of Wannan Medical College), Wuhu, China; 3 The Institutes of Brain Science, Wannan Medical College, Wuhu, China

**Keywords:** apoptosis, cell cycle, glioma, invasion, ITPKA, prognosis, progression, proliferation

## Abstract

**Background:**

Inositol 1,4,5-trisphosphate 3-kinase A (ITPKA) is expressed in various tumors and is associated with tumor progression. This study investigated the expression patterns of ITPKA in gliomas and explored its functional role in glioblastoma (GBM), thereby providing new insights into the diagnostic and prognostic evaluations of ITPKA in this disease.

**Methods:**

ITPKA expression levels in glioma tissues of different World Health Organization (WHO) grades and GBM cell lines were measured using reverse transcription quantitative polymerase chain reaction (RT-qPCR) and Western blotting. U251-MG and T98G cells were transfected with negative control, ITPKA overexpression or ITPKA knockdown plasmids, followed by relevant detections. Subsequently, the viability, proliferation, migration and invasion abilities, cell cycle progression, and apoptosis rate of GBM cells in each group were detected using the Cell-Counting Kit-8 (CCK-8), 5-ethynyl-2′-deoxyuridine (EdU) proliferation, wound healing, Transwell, and flow cytometry assays, respectively. Subsequently, the effect of ITPKA on GBM growth was evaluated in a nude mouse subcutaneous tumor xenograft model.

**Results:**

Overexpression of ITPKA significantly inhibited the proliferation, migration, and invasion capabilities of GBM cells of the two GBM cell lines *in vitro* and the progression of subcutaneous xenograft tumors of two GBM cell lines in nude mice *in vivo*. In contrast, ITPKA knockdown significantly promoted the proliferation, migration, and invasion capabilities of GBM cells in the two GBM cell lines *in vitro* and the progression of subcutaneous xenograft tumors in these two GBM cell lines in nude mice.

**Conclusions:**

Low expression of ITPKA in glioma tissues correlates with GBM progression, indicating that it may act as a tumor suppressor gene and is a candidate biomarker for the molecular diagnostic and prognostic evaluations of glioblastoma.

## 1 Introduction

As the most prevalent primary malignant tumor type of the central nervous system (CNS), gliomas still present a discouraging therapeutic landscape. Statistical analyses have shown that the five-year survival rate of patients with malignant brain tumors is approximately 35%, while the five-year relative survival rate with gliomas is only 7% (1–3). Glioma development is mediated by complex biological processes involving genetic mutations, environmental exposure, and metabolic alterations. Well-established risk factors include high-dose ionizing radiation, chronic inflammation, and family history of glioma (3). Currently, the standard-of-care (SOC) regimen for gliomas consists of surgery combined with radiotherapy and chemotherapy. However, tumors tend to develop drug resistance during treatment, resulting in diminished therapeutic efficacy. Disease recurrence may occur in nearly all patients following SOC (4, 5). Against this backdrop, advances in targeted therapies have provided new hope for the clinical management of gliomas. Recent clinical research has demonstrated that various targeted therapeutic strategies have achieved phase breakthroughs in the field of glioma treatment, and numerous studies have confirmed that such therapeutic approaches can significantly improve progression-free survival (PFS). Notably, several recent studies have suggested that specific targeted treatment regimens have a positive effect on prolonging overall survival (OS) of glioma or GBM patients (6–8). Therefore, an in-depth exploration of potential driver genes regulating gliomas and their molecular mechanisms is crucial to identifying novel therapeutic targets and developing effective targeted molecular therapies, which are of great clinical significance for the effective treatment of glioma.

Three members of the inositol 1,4,5-trisphosphate (InsP_3_) 3-kinase family—InsP_3_ 3-kinase A (ITPKA), InsP_3_ 3-kinase B (ITPKB), and InsP_3_ 3-kinase C (ITPKC)—share a conserved catalytic domain at the C-terminus (9–11). However, these family members exhibit cell- and tissue-specific expression patterns. For instance, ITPKA is predominantly expressed in the brain and testes, with high expression levels detected in the hippocampal pyramidal cells and cerebellar Purkinje cells, while ITPKB is expressed in tissues, including the lungs, thymus, and heart (12, 13). ITPKA has been found to be expressed in various tumor tissues; specifically, its expression level is significantly higher in 19 types of tumors than in non-malignant tissues (14–20). In addition, it has been shown to promote the tumor cell migration of various tumor types (14, 15), including ovarian cancer, renal cell carcinoma, oral squamous cell carcinoma, breast cancer, and hepatocellular carcinoma (16–20). In renal cell carcinoma, breast cancer, and hepatocellular carcinoma, ITPKA overexpression is closely associated with tumor invasion, metastasis, and staging, and directly affects patient prognosis (16–18). In ovarian cancer and oral squamous cell carcinoma, ITPKA expression level is relatively low; however, its activation can inhibit tumor growth and proliferation (18, 20). Taken together, the expression levels of ITPKA vary among different tumor types, indicating that it may serve as a potential prognostic marker.

Previous studies have shown that ITPKA hypomethylation is significantly associated with glioma prognosis, and that this gene is enriched in the phosphatidylinositol metabolic pathway (21). However, the specific expression pattern, functional role, and underlying molecular mechanisms of ITPKA in gliomas remain unclear and warrant further in-depth investigation.

This study aimed to systematically explore the expression patterns, functional roles, and potential molecular mechanisms of ITPKA in glioma. To this end, this study first utilized The Chinese Glioma Genome Atlas (CGGA) database and The Cancer Genome Atlas (TCGA) database to analyze the expression patterns of ITPKA in glioma, correlate its relationship with clinicopathological parameters, and simultaneously explore the prognostic value of ITPKA in the CGGA database. Subsequently, we further detected the expression levels of ITPKA in glioma specimens of different WHO grades in our own collection and glioma cell lines. The results showed that ITPKA was consistently downregulated in glioma specimens in two public glioma datasets, our own collected glioma specimens, and glioma cell lines. We conducted cellular functional experiments in GBM cell lines U251 MG and T98G with upregulation and downregulation of ITPKA gene. We found that ITPKA upregulation and downregulation significantly inhibited and promoted the proliferative, migratory, and invasive abilities of GBM cells *in vitro*, respectively. *In vivo* experiments further confirmed that overexpression of ITPKA had an inhibitory effect on the growth of glioma. In this study, we are the first to show that ITPKA acts as a tumor suppressor gene in glioblastoma (GBM). These findings provide a novel theoretical basis for clarifying the molecular mechanisms through which ITPKA regulates GBM progression, optimizing the molecular diagnostic and prognostic evaluation system for glioma, and providing a novel candidate target for glioma targeted therapy.

## 2 Materials and Methods

### 2.1 Cell culture

Human embryonic kidney 293T cells (HEK 293T), human brain GBM cell lines (U87-MG, U251-MG, and T98G), and a human brain astrocyte cell line (SVGp12) were obtained from the Cell Bank of the Chinese Academy of Sciences (Shanghai, China). All the cells were routinely cultured in Dulbecco’s modified Eagle’s medium (DMEM) (KGL1206-500; KeyGen Biotech, China) supplemented with 10% fetal bovine serum (FBS) (F0193-500ML; Sigma-Aldrich, USA). The cells were incubated in a humidified incubator with an atmosphere of 5% CO_2_ and 95% air at 37 °C.

### 2.2 Clinical samples

This study was approved by the Ethics Committee of the Second Affiliated Hospital of Wannan Medical College. A total of 12 tumor tissue specimens were collected from glioma patients admitted to the Affiliated Hospital of Wannan Medical College in recent years. Additionally, four normal brain tissue specimens were obtained from non-glioma patients who underwent craniocerebral surgery for traumatic or spontaneous intracerebral hemorrhage (22). The four normal brain tissues were harvested from brain regions, such as the temporal lobe, of patients. These normal brain tissues were confirmed without pathological abnormalities on postoperative pathological examination. The diagnosis of glioma in patient specimens was confirmed by experienced pathologists, and gliomas were graded according to the 2021 World Health Organization (WHO) classification of CNS tumors (23). The detailed clinicopathological characteristics of the 12 patients with glioma, including age, sex, histopathological type of glioma, and WHO pathological grade, are summarized in **Supplementary Table 1**. In the present study, for statistical analysis, all the glioma specimens were divided into two subgroups: low-grade gliomas (LGG; WHO grades I - II, n=6) and high-grade gliomas (HGG; WHO grades III - IV, n=6). Fresh intraoperative tissues were immediately snap-frozen in liquid nitrogen for subsequent protein and RNA extractions. Tissues were fixed in formalin and embedded in paraffin for immunohistochemical (IHC) analysis.

### 2.3 Establishment of stable cell lines with ITPKA overexpression or knockdown

Two ITPKA knockdown plasmids, one ITPKA overexpression plasmid, and two control plasmids were supplied by Wuhan Miaoling Biotechnology Co., Ltd. (Wuhan, China) (24). In strict accordance with the manufacturer’s instructions, these plasmids were transfected into human embryonic kidney 293T cells (HEK 293T) for lentivirus packaging to yield five lentiviral constructs. Subsequently, these lentiviruses were transduced into target cells (U251-MG and T98G cells) to establish stable ITPKA overexpression and knockdown cell lines. Puromycin (2 mg/ml; P8230-25mg, purchased from Beijing Solarbio Science & Technology Co., Ltd., China) was added to the lentivirus-infected cells for a continuous 2-week selection, which finally resulted in the establishment of stably transfected cell lines. The plasmid sequences are listed as follows: oe-ITPKA-1: 5’-TCTAGAGCTAGC-3’; sh-ITPKA-1: 5’-GCCGGTCATAAGCCCTTTCAAccctttcaa-3’; sh-ITPKA-2: 5’-CGGAAGGACATGTACAAGAAA’.

### 2.4 Reverse transcription quantitative polymerase chain reaction analysis

The total RNA was extracted from normal human brain tissue samples, human GBM tissue samples, and cell lines (U87-MG, T98G, U251-MG, and SVGp12) using the TRIzol reagent (15596026CN; Thermo Fisher Scientific, USA) according to the manufacturer’s protocol (24). The total RNA was reverse-transcribed into complementary DNA (cDNA) using a FastKing RT Kit (KR116; TianGen Biotech (Beijing) Co., Ltd., China). The RT-qPCR primers were as follows: ITPKA: 5’-GGACGTGGGTCAGAAAAACC-3’ (forward) and 5’-TAAAACTCCCAGTGTGCCCT-3’ (reverse); GAPDH: 5’-GGAAGCTTGTCATCAATGGAAATC-3’ (forward) and 5’-TGATGACCCTTTTGGCTCCC-3’ (reverse). *RT‐qPCR* analysis was performed on an ABI 7500 Real-Time PCR System (Applied Biosystems, USA) using SuperReal PreMix Plus SYBR Green reagent (FP205; TianGen Biotech (Beijing) Co., Ltd., China).

### 2.5 Western blotting

Western blotting was used to detect ITPKA expression in normal human brain tissue samples, human glioma tissue samples, and GBM cell lines (U87-MG, T98G, U251-MG, and SVGp12) (22, 24, 25). Protein samples were prepared using radioimmunoprecipitation assay buffer (RIPA) (P0013E-1; Beyotime Biotechnology, China) containing 1% protease inhibitors. The samples were quantified using the BCA (P0010; Beyotime Biotechnology, China) method. Subsequently, each sample with an equal amount of total protein was mixed with a loading buffer (P0015L; Beyotime Biotechnology, China) and heated at 100 °C for 10 minutes. Proteins were separated using a 10% Color PAGE Gel Rapid Preparation Kit (PG112; Shanghai Yazyme Biopharmaceutical Technology Co., Ltd, China) and then electrotransferred from the gel to a PVDF membrane (IPVH00010; Millipore, USA). The membrane was incubated with blocking buffer (P0260; Beyotime Biotechnology, China) on a shaker platform for 30 minutes at room temperature and then incubated with a primary antibody at 4 °C overnight (ITPKA Polyclonal antibody, 1:1000; 14270-1-AP; Proteintech, China). After thorough washing, the membrane was incubated with a secondary antibody (anti-rabbit immunoglobulin G, 1:10,000; Abs20040; Absin Bioscience, China) at room temperature for 1 hour. After another round of thorough washing, the expression level of ITPKA was detected using a chemiluminescence kit (WBKLS; Millipore, USA).

### 2.6 Immunohistochemistry

The expression levels of ITPKA and Ki-67 in normal brain tissues, LGGs and HGGs were detected by immunohistochemical (IHC) staining (22, 24). Paraffin-embedded tissue sections were dewaxed in xylene and rehydrated in a graded series of ethanol prior to routine IHC staining. For antigen retrieval, the sections were immersed in 0.1 M citrate buffer (pH 6.0) and steamed for 15 minutes in a steamer. Subsequently, a 3% hydrogen peroxide (H_2_O_2_) solution was added dropwise to the tissue sections, which were then incubated in the dark for 10 minutes to block endogenous peroxidase activity. Nonspecific binding was blocked using phosphate-buffered saline (PBS) containing 5% bovine serum albumin (BSA) at room temperature for 30 minutes. The sections were then incubated overnight at 4 °C with each of the primary antibodies, including anti-ITPKA monoclonal antibody (1:200; 14270-1-AP, Proteintech Group, Wuhan, China) and anti-Ki-67 polyclonal antibody (1:8000; 27309-1-AP, Proteintech Group, Wuhan, China). After thorough rinsing with PBS, secondary antibody staining was performed using an IHC kit (Kgos60; Keygen Biotech, Nanjing, China) according to the manufacturer’s instructions. Nuclei in tissue sections were counterstained with a hematoxylin solution. Subsequently, the tissue sections were dehydrated in a graded series of ethanol, cleared in xylene, and coverslipped. Finally, the tissue sections were observed and photographed under an upright microscope (Axio Scope A1; Carl Zeiss, Germany).

### 2.7 Cell proliferation assay

The Cell-Counting Kit-8 (CCK-8) assay was used to detect the effect of ITPKA overexpression and knockdown on the proliferation of U251-MG and T98G cells (22, 24, 25). Stably transfected U251-MG or T98G cells were seeded into 96-well cell culture plates at a density of 4000 cells per well. After 1 hour of cell culture (to allow cell adhesion), 10 µL of the CCK-8 solution (BS350B; Biosharp, China) was added to each well. Following a one-hour incubation, the absorbance was measured at 450 nm using a microplate reader (BioTek, USA). Subsequently, the CCK-8 assay was performed on the cells every 24 hours for four consecutive days, and cell proliferation curves were drawn.

### 2.8 Cell migration and invasion assay (Transwell assay)

The effects of ITPKA overexpression and knockout on the migration and invasion capabilities of cells (U251-MG and T98G) were determined using a Transwell assay (22, 24, 26). Chambers with Transwell inserts in 24-well plates (pore size: 8 μm) (CLS3422; Corning Incorporated, USA) were used. Stably transfected U251-MG and T98G cells were suspended in 200 μL of serum-free DMEM medium at a density of 1×10^5^ cells per well and seeded into the upper chamber. Next, 700 μL of DMEM containing 10% FBS was added to the lower chamber. For the invasion assay, an additional step was required: prior to cell seeding, Matrigel (ABW0827065; ABW, China) was diluted with serum-free DMEM medium at a volume ratio of 1:9 and then uniformly spread on the upper chamber for coating. After 48 hours of incubation, the cells were fixed with 4% paraformaldehyde (BL539A; Biosharp, China) for 20 minutes and then stained with 1% crystal violet staining solution (110703013; Bikemam Bio, China) for 30 minutes. Subsequently, the 1% crystal violet staining solution was recovered, the Transwell inserts were washed, and the cells on the upper surface of the Transwell inserts were wiped off using a cotton swab. The Transwell inserts were carefully cut out and placed on glass slides, and the cells on the lower surface of the Transwell inserts were observed and photographed under an upright microscope (Carl Zeiss Axio Scope A1, Germany). The number of migrated and invaded cells was quantified and analyzed using the ImageJ software.

### 2.9 Cell cycle assay

Flow cytometry was used to detect the effects of ITPKA overexpression and knockdown on cell cycle progression (24). Stably transfected U251-MG and T98G cells were seeded into 6-well cell culture plates at a density of 5×10^5^ cells per well. After 24 hours of culture, the cells were collected into 1 mL of 70% ethanol and fixed overnight at -20 °C. Cells were processed using a cell cycle detection kit (KGA9101-50; Keygen Biotech, Nanjing, China). Cell cycle assay was performed using flow cytometry (Navios, Beckman, USA), and data analysis of cell cycle assay was conducted using ModFit software (ModFit LT 5.0) (Verity Software House, USA).

### 2.10 Cell apoptosis assay

Flow cytometry was used to determine the effects of ITPKA overexpression and knockdown on cell apoptosis (22, 24). A total of 5×10^5^ stably transfected U251-MG and T98G cells were centrifuged, after which apoptosis detection was performed using the Annexin V-APC/PI Double Staining Apoptosis Detection Kit (KGA1107-100; Keygen Biotech, Nanjing, China). The centrifuged cell pellets were resuspended in a 500 μL of the binding buffer, followed by the addition of 5 μL of Annexin V-APC solution and 5 μL of propidium iodide (PI) solution. The mixture was then incubated for 20 minutes at room temperature in the dark. The cell apoptosis assay was performed using a flow cytometer (Navios, Beckman, USA), and data analysis of the cell apoptosis assay was conducted using FlowJo software (FlowJo 10.8.1) (Becton, Dickinson & Company, USA).

### 2.11 Xenograft tumor inhibition mouse model

BALB/c nude male mice (6–8 weeks old, weighing 18–22 g) were purchased from Jiangsu Qinglongshan Biotechnology Co., Ltd. (Nanjing, China) (License No.: SCXK (Su) 2024-0001). The mice were housed in a specific pathogen-free (SPF)-grade animal facility with free access to standard chow and sterile water. All animal experiments were performed in accordance with the Guidelines for the Care and Use of Laboratory Animals of China and were approved by the Institutional Animal Care and Use Committee (IACUC) of the Second Affiliated Hospital of Wannan Medical College. Stably transfected U251-MG and T98G cells (5×10^6^ cells/mL), including the control and overexpression groups (oe-NC, oe-ITPKA-1) as well as the control and knockdown groups (sh-NC, sh-ITPKA-1, sh-ITPKA-2), were resuspended and then subcutaneously injected into the right flank of each BALB/c nude mouse for *in vivo* tumorigenicity assays (22, 25, 27), with three mice allocated to each experimental group. On the 45th day post-injection, the xenograft tumors were excised, weighed immediately, and stored for subsequent analysis.

### 2.12 Statistical analysis

Experimental data are presented as mean ± standard deviation. All data were statistically analyzed using GraphPad Prism 8 (GraphPad Software, Boston, MA, USA). For comparisons between two groups, statistical differences were analyzed using a two-tailed Student’s t-test. For comparisons involving more than two groups, one-way analysis of variance (ANOVA) followed by multiple comparison tests was applied. Differences were considered statistically significant when the values of p are less than 0.05.

## 3 Results

### 3.1 Downregulation of ITPKA in glioma in two public glioma datasets and its clinical significance

**Figure 1:**
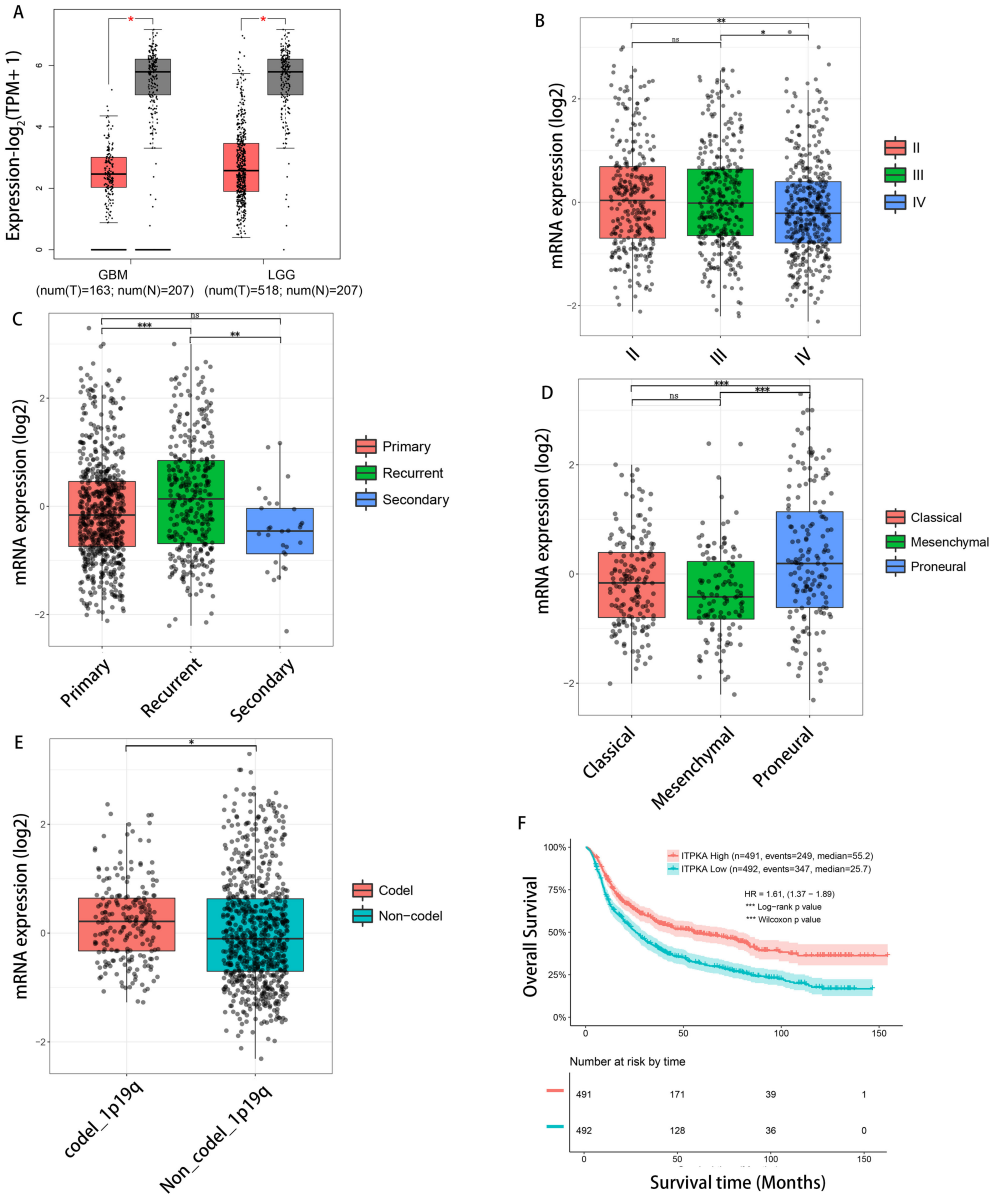
The expression level of ITPKA gene in glioma tissues within the Chinese Glioma Genome Atlas (CGGA) database is closely related to the survival and prognosis of patients. **(A)** shows the relative expression differences of ITPKA gene mRNA between the tumor group and the normal group in the GBM (GBM) and Low-Grade Glioma (LGG) databases of The Cancer Genome Atlas (TCGA), respectively. **(B)** shows the relative expression differences of ITPKA gene mRNA in patients with World Health Organization (WHO) grade II, III, and IV gliomas in the CGGA database. **(C)** shows the relative expression differences of ITPKA gene mRNA in patients with primary, recurrent, and secondary gliomas in the CGGA database. **(D)** shows the relative expression differences of ITPKA gene mRNA in patients with classical, mesenchymal, and proneural gliomas in the CGGA database. **(E)** shows the relative expression differences of ITPKA gene mRNA in glioma patients with 1p/19q chromosome codeletion and non-codeletion in the CGGA database. **(F)** shows the correlation between the mRNA expression level of ITPKA gene and the survival time of glioma patients in the CGGA database. Data presented as mean ± SD; ns: not significant; *p < 0.05, **p < 0.01, ***p < 0.001. T: tumor tissue; N: normal tissue; num: number.

We evaluated the ITPKA expression level in the TCGA glioma dataset using the GEPIA2 database (http://gepia2.cancer-pku.cn) and found that the transcriptional level of ITPKA in high-grade gliomas (HGGs) and low-grade gliomas (LGGs) was significantly lower than that of normal tissues (**Figure 1A**). Subsequently, we utilized GlioVis (https://gliovis.bioinfo.cnio.es/), a visualization and analysis tool for brain tumor expression databases, to investigate the correlation between ITPKA expression and various clinicopathological features of gliomas in the CGGA database. Our results showed that ITPKA expression was slightly but significantly reduced in WHO grade IV gliomas compared to that in WHO grade II and III gliomas (**Figure 1B**). Regarding tumor progression, the ITPKA expression levels in patients with recurrent gliomas were remarkably higher than those in patients with primary and secondary gliomas (**Figure 1C**). In terms of the expression differences in molecular subtypes, the ITPKA expression in patients with proneural gliomas was significantly higher than that in patients with classical and mesenchymal gliomas (**Figure 1D**). In relation to chromosomal variations, ITPKA was overexpressed in the tissues of patients with 1p/19q codeletion compared to patients without 1p/19q codeletion (**Figure 1E**).

Glioma patients were divided into low-expression and high-expression groups based on the median expression level of ITPKA and then survival analysis of these patients was conducted using the CGGA database. Notably, we observed that the overall survival (OS) of patients with low ITPKA expression was significantly shorter than that of patients with high ITPKA expression (**Figure 1F**), indicating that ITPKA is a negative prognostic factor for glioma patients.

### 3.2 Consistent downregulation of ITPKA expression in glioma tissues of our collection and GBM cell lines

**Figure 2:**
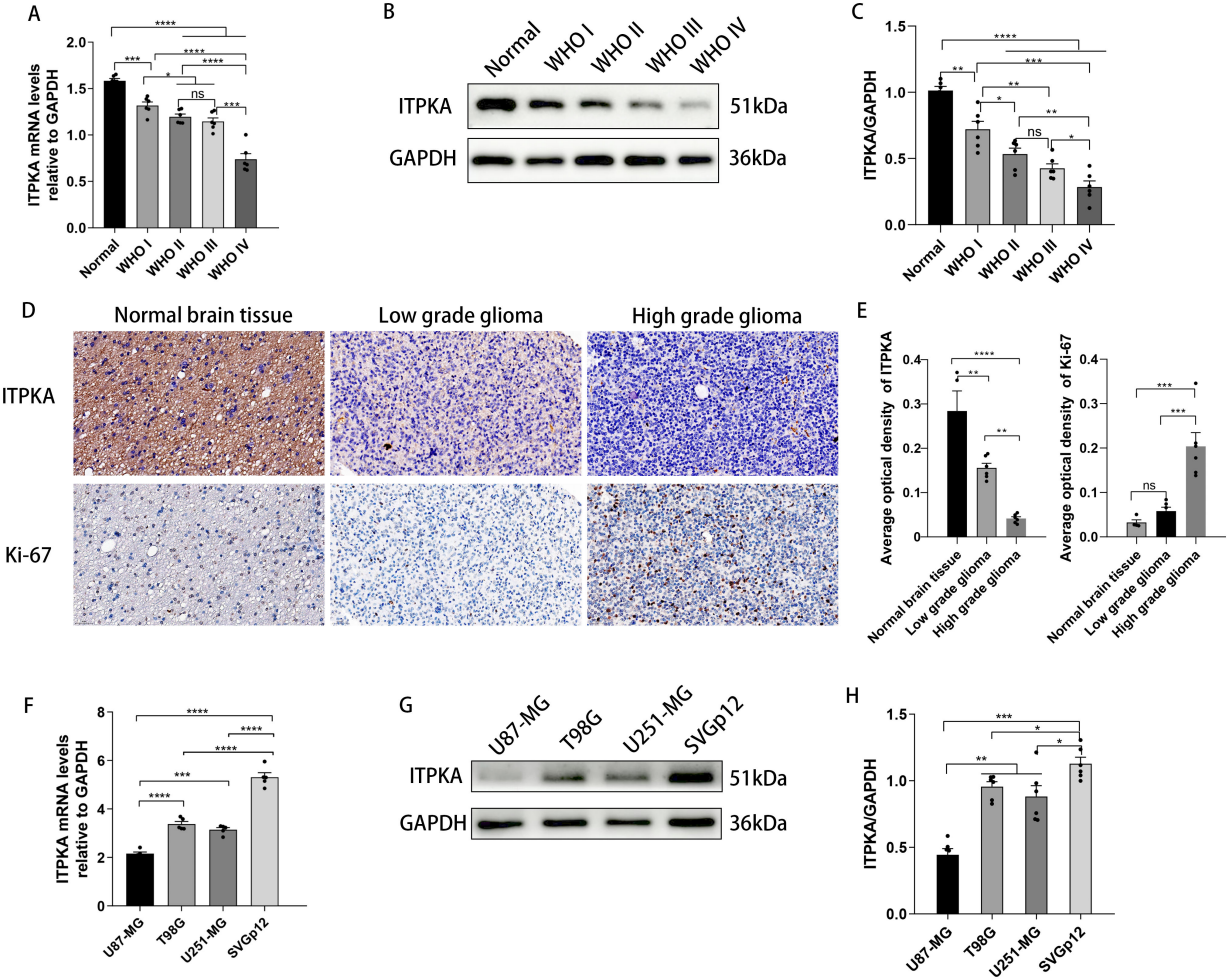
Validation of ITPKA expression in glioma patient specimens of our collection and GBM cells. **(A-C)** RT-qPCR **(A)** or Western blotting **(B, C)** shows the relative expression difference of ITPKA between normal brain tissues and glioma tissues of different grades in our collection of glioma patient samples. **(D)** Immunohistochemical staining (IHC) shows the representative images of ITPKA and Ki-67 proteins in normal brain tissues, low-grade glioma and high-grade glioma tissue specimens (under ×40 magnification, Scale bar = 50 µm). **(E)** Immunohistochemical staining (IHC) presents the quantitative analysis of the average optical density of ITPKA and Ki-67 in normal brain, low-grade glioma and high-grade glioma tissue specimens. **(F-H)** RT-qPCR **(F)** and Western blotting **(G, H)** show the relative expression difference of ITPKA in human normal astrocyte cell line, SVGp12 and three indicated GBM cell lines. Data presented as mean ± SD; the Student’s t-test and one-way analysis of variance (ANOVA) followed by multiple comparison tests were conducted. The experiments were independently repeated at least three times. ns: not significant; *p < 0.05, **p < 0.01, ***p < 0.001, ****p < 0.0001. Normal: normal brain tissues.

To evaluate the expression levels of ITPKA in tissue samples from glioma patients that we collected, we performed RT-qPCR and Western blotting assays. RT-qPCR results revealed that compared with normal human brain tissues, the level of ITPKA mRNA decreased as the glioma grade increased (**Figure 2A**). Consistently, representative images from Western blotting analysis showed that the protein level of ITPKA decreased with the increasing glioma grade (**Figures 2B, C**). To ensure the reliability of the above findings, we performed immunohistochemical (IHC) staining of tissue samples from the same cohort of glioma patients. IHC analysis confirmed that the expression level of ITPKA was significantly higher in LGGs than in HGGs, whereas the expression of the cell proliferation marker, Ki-67, was markedly elevated in HGGs (**Figure 2D, E**).

To examine the expression level of ITPKA at the cellular level, we performed RT-qPCR and Western blotting assays on three GBM cell lines (U87-MG, T98G, and U251-MG) and one immortalized human normal astrocyte cell line (SVGp12). RT-qPCR results demonstrated that the expression level of ITPKA mRNA was significantly downregulated in all three GBM cell lines compared with that in SVGP12 (**Figure 2F**). Consistently, Western blotting experiments showed that ITPKA levels were significantly decreased in all three GBM cell lines, compared with that in SVGp12 (**Figures 2G, H**).

### 3.3 Effect of ITPKA overexpression on GBM cells

**Figure 3:**
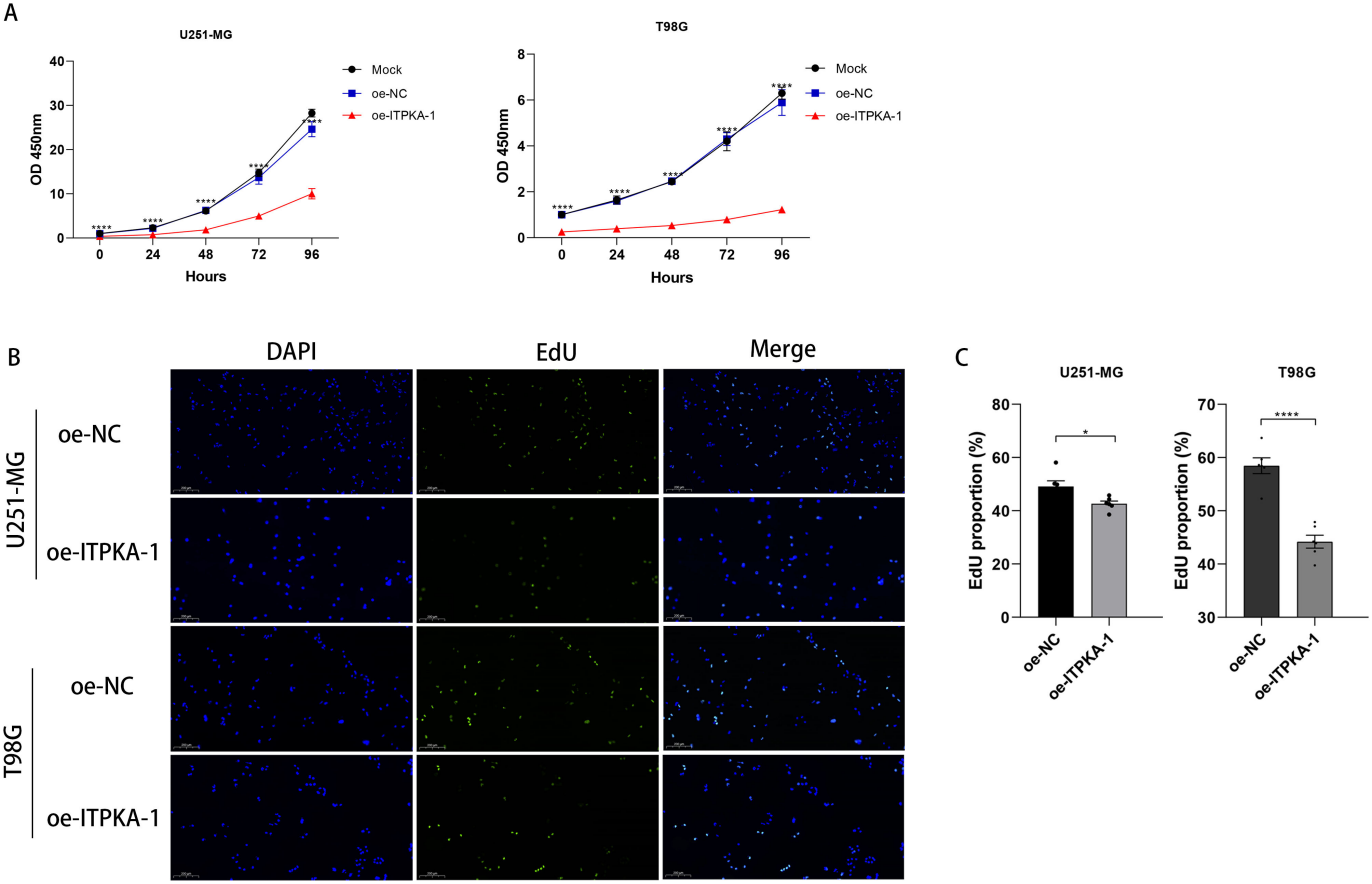
Effect of ITPKA overexpression on the functional characteristics of GBM cells. **(A)** CCK-8 proliferation assay was performed in U251-MG and T98G cells; **(B, C)** EdU assay was conducted in U251-MG and T98G cells (under ×10 magnification, Scale bar = 200 µm). All experimental data are presented as mean ± standard deviation (SD) from six independent repeated experiments, and Student’s t-test was used for statistical analysis. Data annotation: ns: not significant; *p < 0.05, ****p < 0.0001.

After we successfully established stable ITPKA-overexpressing (oe-ITPKA) U251-MG and T98G cell lines (**Supplementary Figure 1**), we investigated the effect of ITPKA overexpression on some of the malignant biological behaviors of GBM cells. First, to verify the regulatory role of ITPKA in the proliferative capacity of GBM cells, the Cell Counting Kit-8 (CCK-8) and 5-ethynyl-2′-deoxyuridine (EdU) proliferation assays were employed. The results showed that ITPKA overexpression significantly inhibited the proliferative capabilities of both U251-MG and T98G cells in the CCK-8 assay (**Figure 3A**) and EdU assay (**Figures 3B, C**).

**Figure 4:**
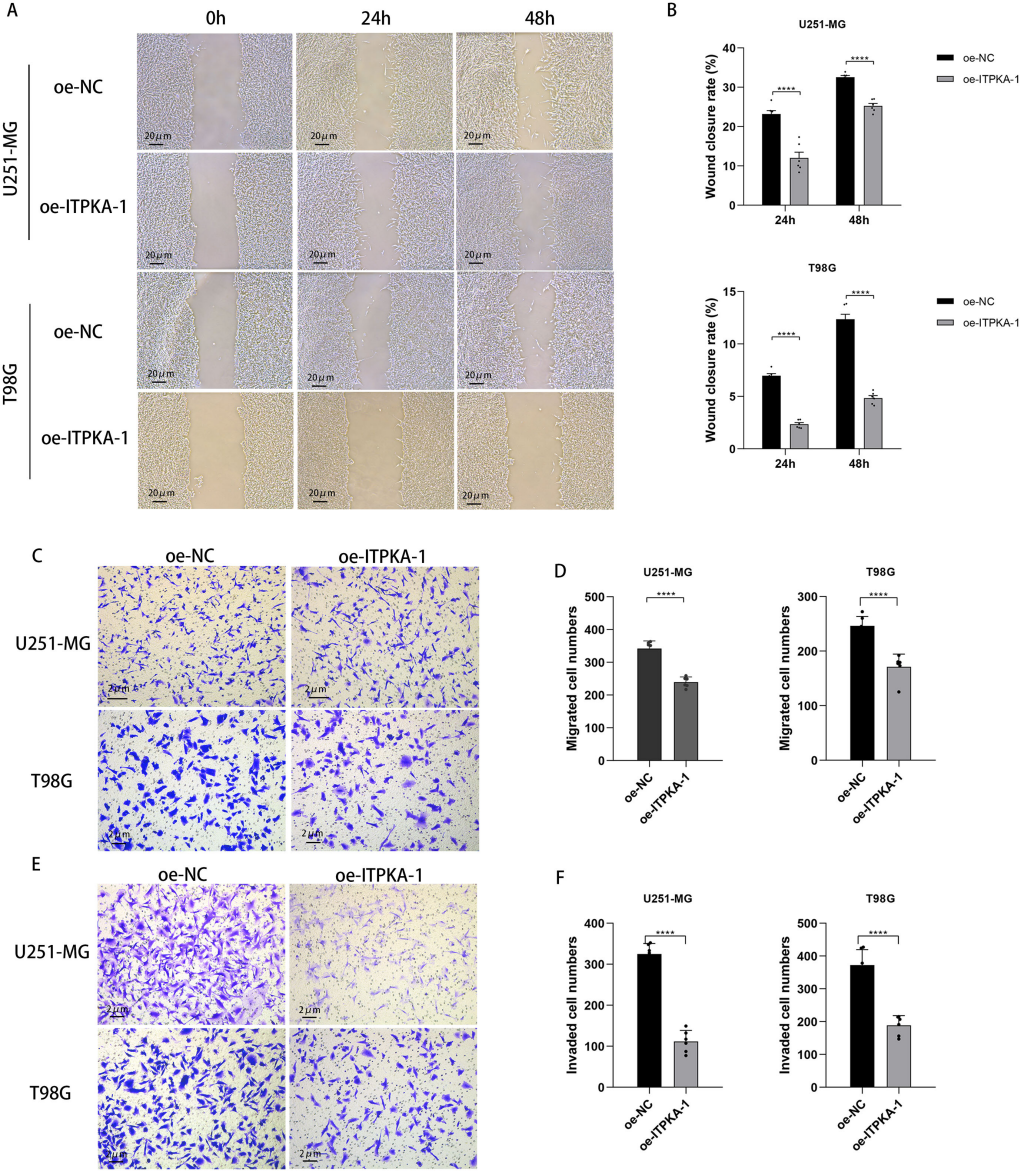
Effect of ITPKA overexpression on the functional characteristics of GBM cells. **(A, B)** Wound healing assay was performed in U251-MG and T98G cells (under ×20 magnification, Scale bar = 20 µm); **(C-F)** Transwell assay was conducted in U251-MG and T98G cells (under ×200 magnification, Scale bar = 2 µm). All experimental data are presented as mean ± standard deviation (SD) from six independent repeated experiments, and Student’s t-test was used for statistical analysis. Data annotation: ns: not significant; ****p < 0.0001.

To investigate the effect of ITPKA overexpression on the migration and invasion capabilities of GBM cells, we conducted scratch wound healing and Transwell assays. In the scratch wound healing assay, the wound closure rate of the oe-ITPKA group (oe-ITPKA-1) was significantly lower than that of the control group (oe-NC) (**Figures 4A, B**). In the Transwell assay, the number of migrating and invading cells in the oe-ITPKA-1 group was significantly lower than that of the control group (oe-NC) (**Figure 4C–F**).

**Figure 5:**
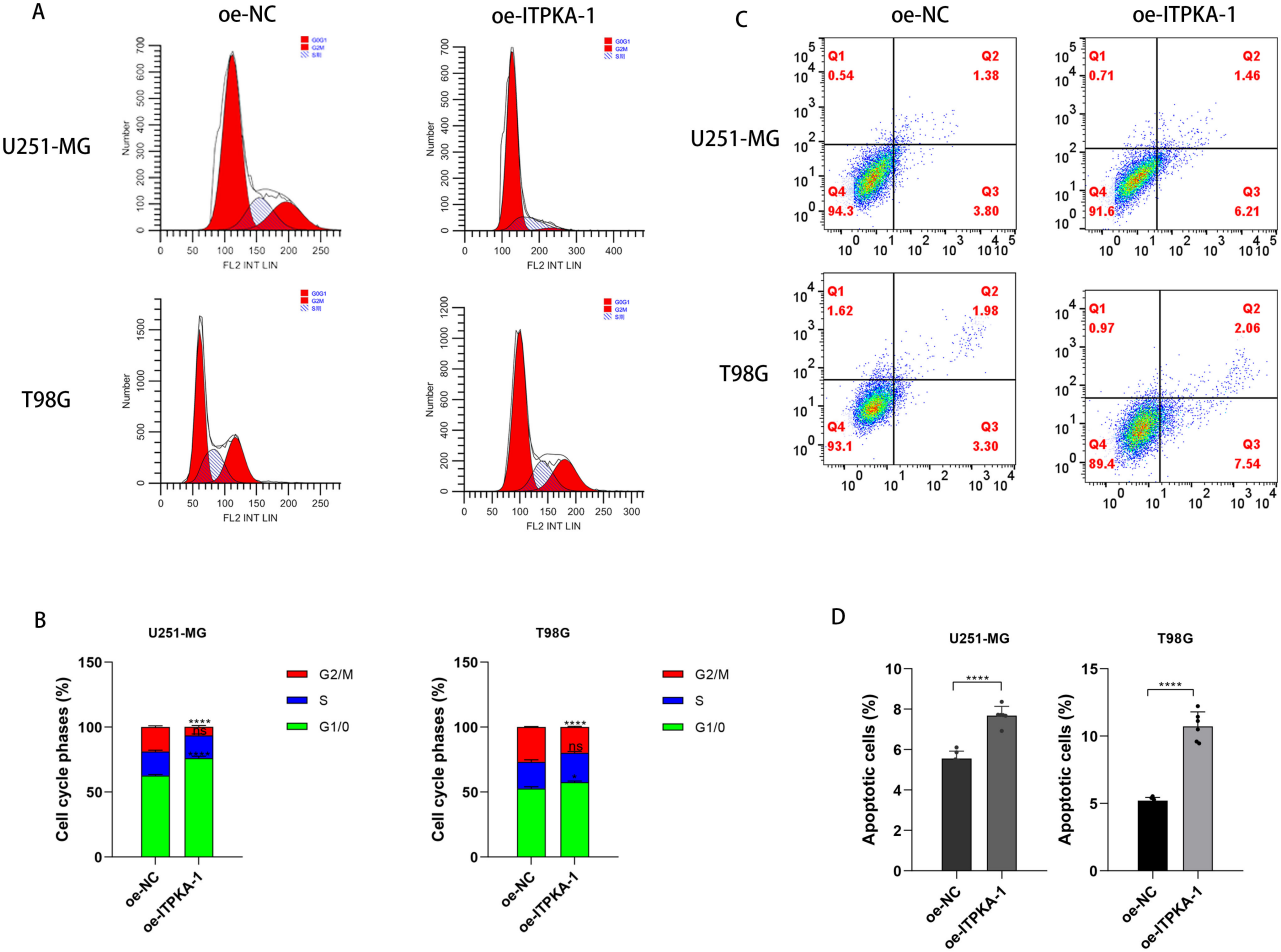
Effect of ITPKA overexpression on the functional characteristics of GBM cells. Flow cytometry analysis **(A, B)** shows the effect of upregulated ITPKA expression on the cell cycle progression of U251-MG and T98G cells; **(C, D)** evaluates the effect of upregulated ITPKA expression on the apoptosis level of the two indicated cell lines. All experimental data are presented as mean ± standard deviation (SD) from six independent repeated experiments, and Student’s t-test was used for statistical analysis. Data annotation: ns: not significant; *p < 0.05, ****p < 0.0001.

Since ITPKA overexpression significantly inhibited cell proliferation, we examined cell cycle progression as a potential underlying molecular mechanism. Flow cytometry assay showed that ITPKA overexpression significantly arrested cells at the G1 phase of the cell cycle, preventing cells from entering the mitotic phase and thereby inhibiting the cell proliferation rate (**Figures 5A, B**). Cell apoptosis acts as a countermeasure mechanism that regulates tumor growth; therefore, we investigated the effect of ITPKA level on GBM cell apoptosis. The results showed that overexpression of ITPKA significantly increased the apoptotic rate of U251-MG and T98G cells (**Figures 5C, D**).

In conclusion, ITPKA can inhibit the proliferation, migration, and invasion capabilities of GBM cells, while ITPKA inhibits apoptosis of GBM cells, thus exerting a significant tumor-suppressive effect.

### 3.4 Impact of ITPKA silencing on GBM cells

**Figure 6:**
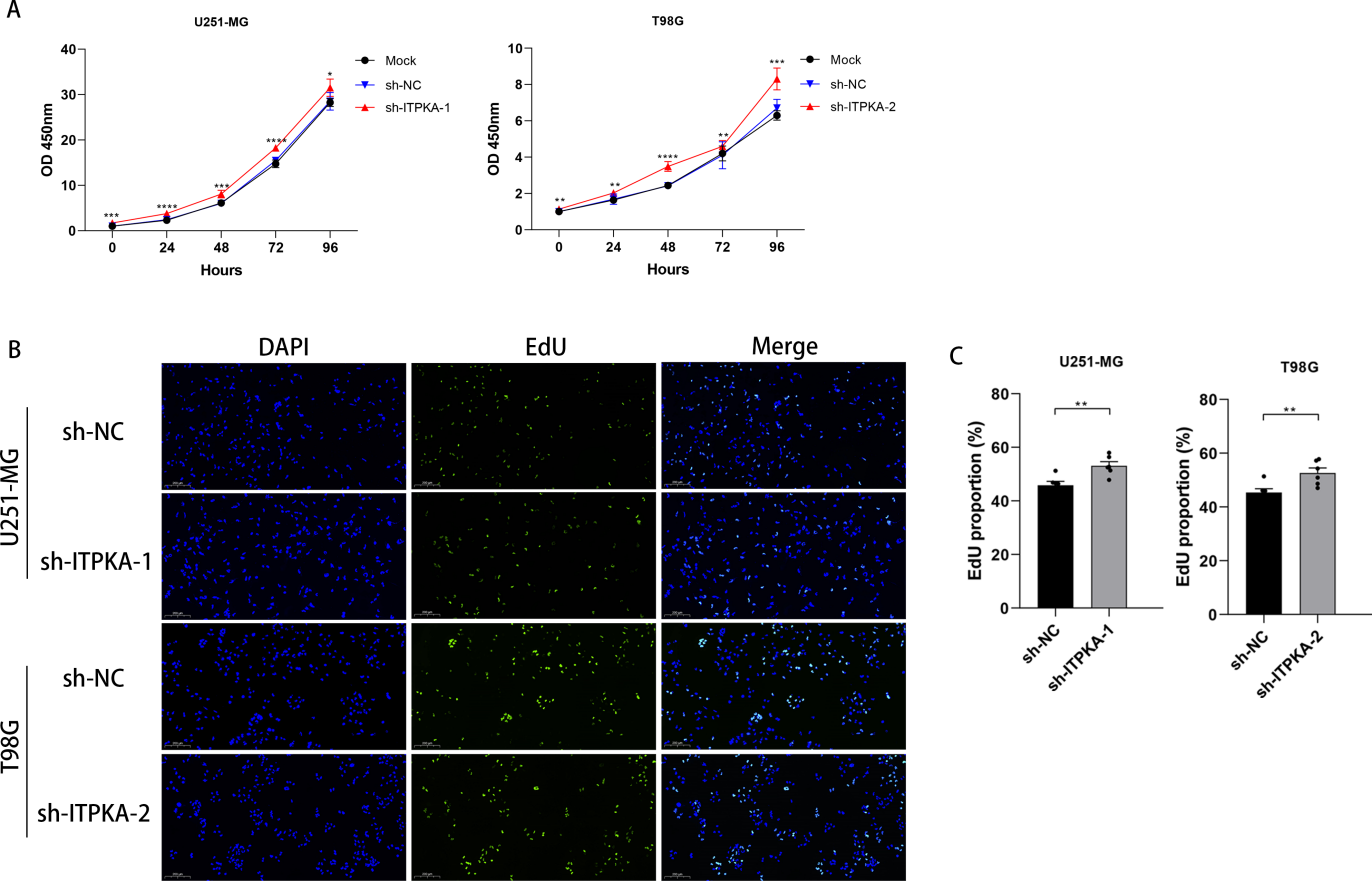
Effect of ITPKA knockdown on the functional characteristics of GBM cells. **(A)** CCK-8 proliferation assay was performed in U251-MG and T98G cells; **(B, C)** EdU assay was conducted in U251-MG and T98G cells (under ×10 magnification, Scale bar = 200 µm). All experimental data are presented as mean ± standard deviation (SD) from six independent repeated experiments, and Student’s t-test was used for statistical analysis. Data annotation: ns: not significant; *p < 0.05, **p < 0.01, ***p < 0.001, ****p < 0.0001.

After we successfully established ITPKA-knockdown (sh-ITPKA) U251-MG and T98G stable cell lines (**Supplementary Figure 1**), we sought to systematically examine the regulatory role of ITPKA in the same malignant behaviors of GBM cells that we previously examined in ITPKA-overexpressing GBM cells. We observed phenotypic differences between the ITPKA-knockdown groups (sh-ITPKA-1 and sh-ITPKA-2) and the negative control group (sh-NC) in GBM cell lines. To explore the effect of ITPKA on GBM cell proliferation and its regulatory mechanism, we performed CCK-8 and EdU proliferation assays. The experimental results showed that in both the U251-MG and T98G cell lines, the cell proliferation capability of the ITPKA knockdown groups was significantly enhanced compared with that of the sh-NC control group in the CCK-8 proliferation assay (**Figure 6A**) and EdU proliferation assay (**Figures 6B, C**).

**Figure 7:**
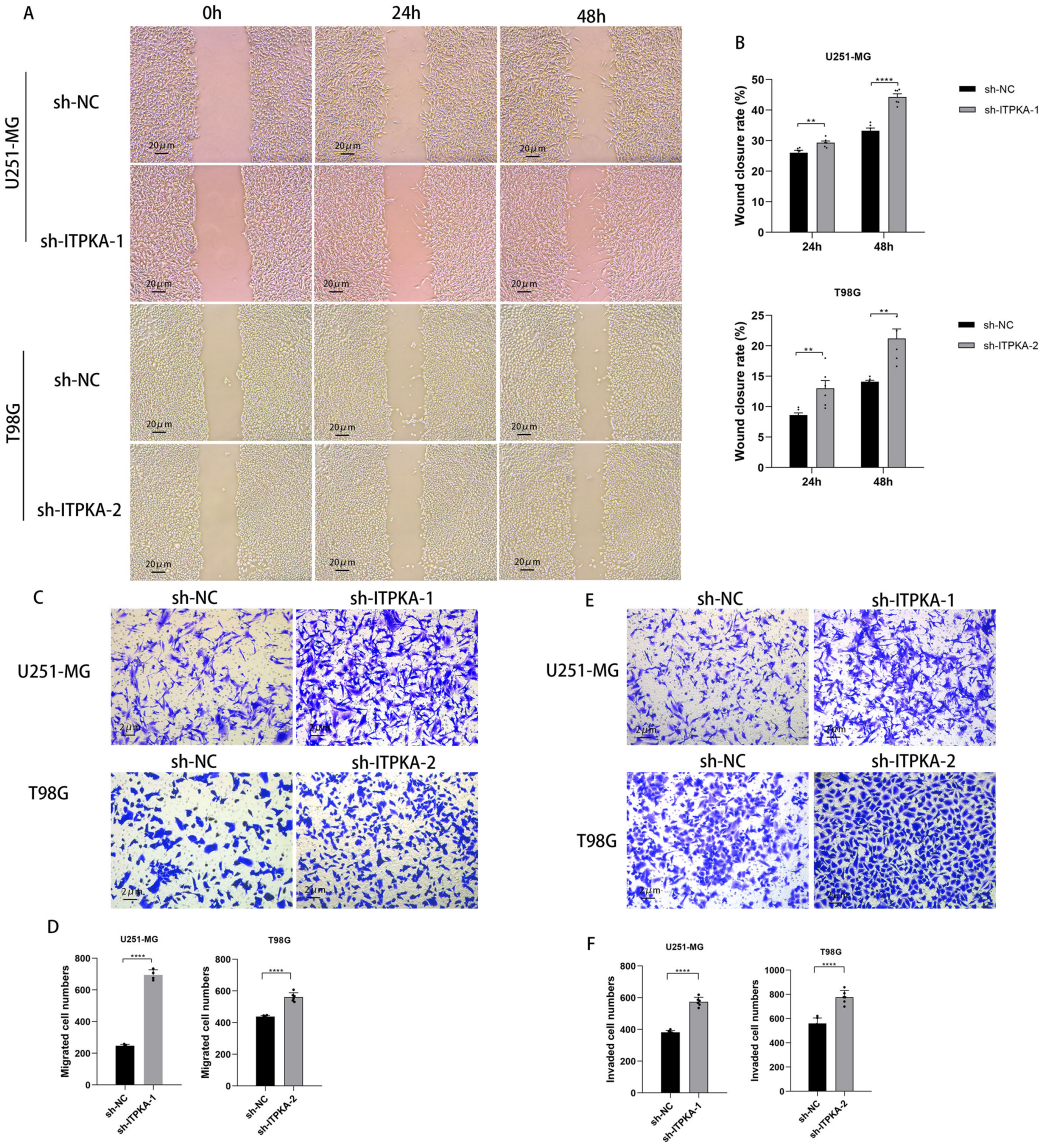
Effect of ITPKA knockdown on the functional characteristics of GBM cells. **(A, B)** Wound healing assay was performed in U251-MG and T98G cells (under ×20 magnification, Scale bar = 20 µm); **(C, F)** Transwell assay was conducted in U251-MG and T98G cells (under ×200 magnification, Scale bar = 2 µm). All experimental data are presented as mean ± standard deviation (SD) from six independent repeated experiments, and Student’s t-test was used for statistical analysis. Data annotation: ns: not significant; **p < 0.01, ****p < 0.0001.

Next, to evaluate the regulatory role of ITPKA in the migration and invasion behaviors of GBM cells, we performed scratch wound healing and Transwell assays. The results of the scratch wound healing assay showed that the healing capability of the ITPKA-knockdown groups at 24 and 48 hours after scratching the wound was significantly higher than that of the sh-NC control group (**Figures 7A, B**). In the Transwell assay, we observed that the number of stained cells that migrated through the insert membrane in the Transwell chamber in the ITPKA-knockdown group was significantly higher than that in the sh-NC control group (**Figures 7C, D**). Similarly, we observed that the number of stained cells that invaded the pre-coated Matrigel barrier layer in the Transwell chamber in the ITPKA-knockdown groups was significantly higher than that in the sh-NC control group (**Figures 7E, F**). These results indicate that ITPKA knockdown promotes the migration and invasion of GBM cells.

**Figure 8:**
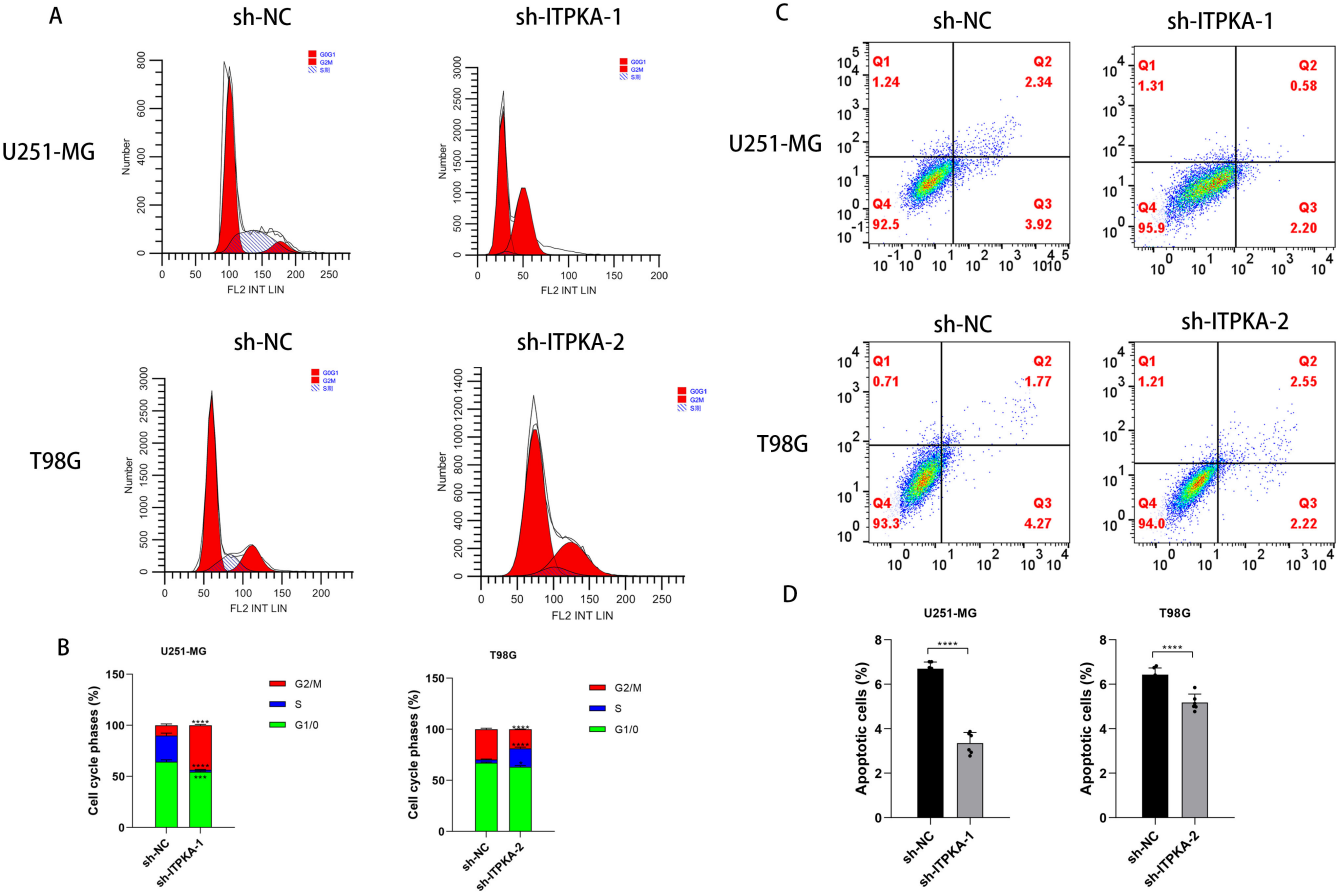
Effect of ITPKA knockdown on the functional characteristics of GBM cells. Flow cytometry analysis **(A, B)** shows the effect of downregulated ITPKA expression on the cell cycle progression of U251-MG and T98G cells; **(C, D)** evaluates the effect of downregulated ITPKA expression on the apoptosis level of the two indicated cell lines. All experimental data are presented as mean ± standard deviation (SD) from six independent repeated experiments, and Student’s t-test was used for statistical analysis. Data annotation: ns: not significant; *p < 0.05, **p < 0.01, ****p < 0.0001.

Finally, to explore the mechanism by which ITPKA regulates the malignant behavior of GBM cells from the key nodes of cell fate regulation, we conducted cell cycle and cell apoptosis assays. The results show that ITPKA silencing reduced the proportion of cells in the quiescent phase (**Figures 8A, B**), inhibited the apoptotic rate of GBM cells, and decreased the number of dead cells (**Figures 8C, D**).

Taken together, these experiments confirm that downregulation of ITPKA expression level can significantly enhance the malignant biological behaviors of GBM cells.

### 3.5 Overexpression of ITPKA inhibits tumor growth of subcutaneous glioblastoma xenografts

**Figure 9:**
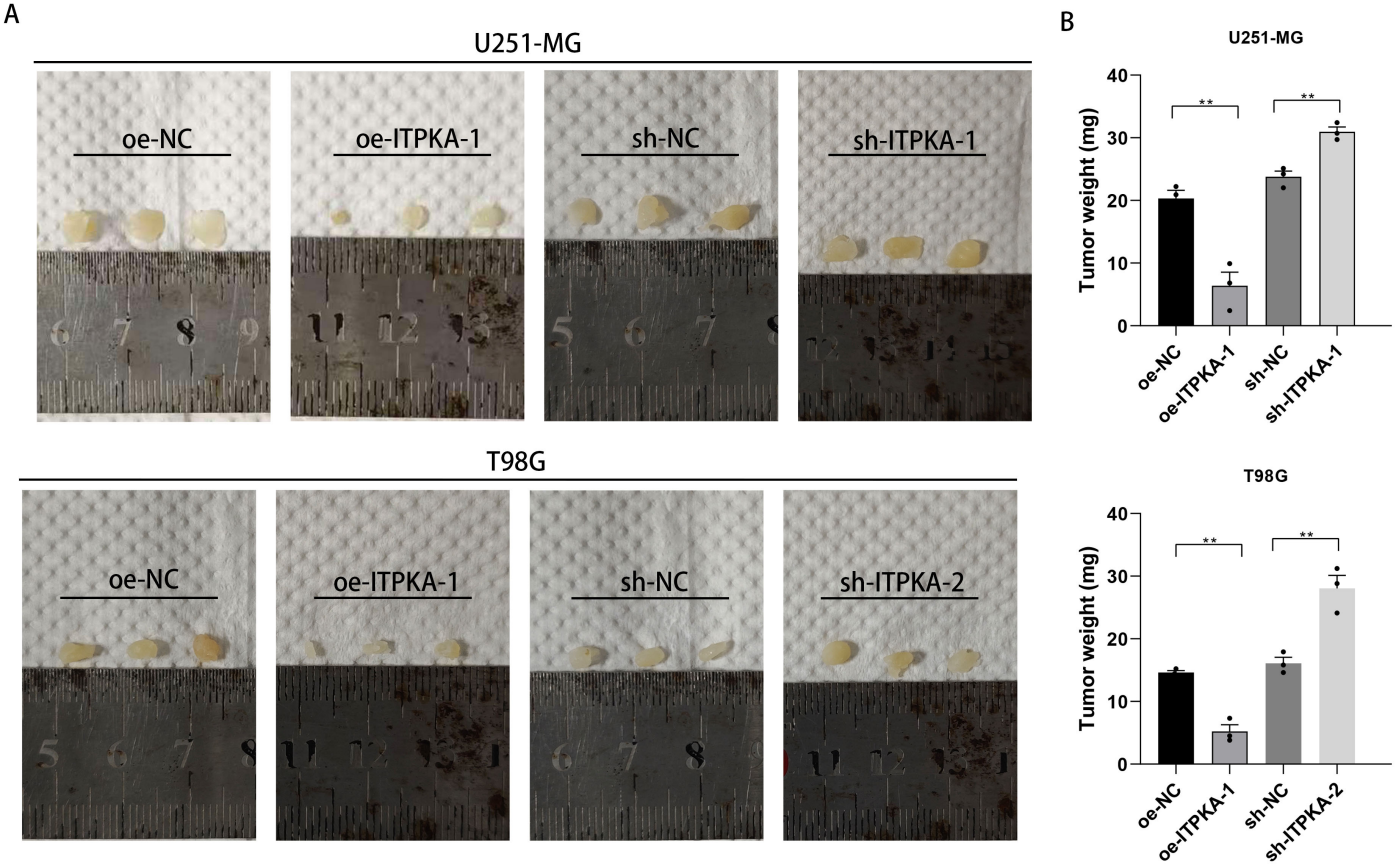
Effect of ITPKA upregulation or downregulation in U251-MG and T98G cells on tumorigenicity and growth in mice. Images of excised subcutaneous tumors in mice **(A)** and statistical analysis of tumor weight **(B)** (n=3). All experimental data are presented as mean ± standard deviation (SD) from three independent repeated experiments, and Student’s t-test was used for statistical analysis. Data annotation: ns: not significant; **p < 0.01.

To further investigate the regulatory role and the mechanism of action of ITPKA in tumorigenesis and tumor progression of GBM cells *in vivo*, we established a subcutaneous xenograft GBM model in nude mouse model. We carefully prepared four cell groups: the ITPKA knockdown group, the ITPKA overexpression group, and their corresponding control groups. Subsequently, these four cell lines were subcutaneously inoculated into the right flank of BALB/c nude mice, providing a stable environment for tumor growth. Mice were euthanized on day 45 after inoculation, and tumor weight in each mouse was accurately measured immediately after sacrifice. The tumor weight was significantly lower in the ITPKA overexpression group than that of its control group, while the tumor weight was markedly higher in the ITPKA-knockdown group than that of its control group (**Figure 9**). Consistent with our *in vitro* findings, the present *in vivo* study confirm that high expression level of ITPKA *in vivo* suppresses GBM growth and progression, whereas low expression level of ITPKA *in vivo* promotes them.

## 4 Discussion

Glioma is the most common primary malignant tumor type of the CNS and is associated with a low survival rate (28). Although standard treatments can improve therapeutic outcomes, the prognosis remains poor (29). Notably, gliomas possess complex genetic and epigenetic mechanisms, and with advances in sequencing technologies, a more in-depth understanding of their significant inter-individual heterogeneity has been achieved (30). Therefore, tailoring individualized treatment regimens based on the unique molecular characteristics of tumors for precise patient therapy has attracted extensive attention in both the academic and clinical fields. This approach can not only significantly delay disease progression but also improve the survival rate and quality of life of glioma patients by accurately identifying eligible patients (29, 31–33).

To date, researchers have identified certain functional abnormalities in multiple tumor suppressor genes in gliomas, such as RB1, PTEN, and TP53 (34, 35). A single RB1 abnormality cannot induce glioma; however, disruption of its related pathways promotes the malignant progression of glioma. In addition, the deletion or mutation of PTEN activates multiple pro-cancer signaling axes to drive abnormal proliferation and malignant transformation of tumor cells. TP53 mutation has clinical specificity, occurs at a high frequency in metastatic gliomas, and can enhance the survivability of tumor cells outside the CNS (36–38). These three factors affect the occurrence, development, and malignant progression of glioma. Beyond the aforementioned known tumor suppressor genes, our study reveals that ITPKA may play a role as a novel tumor suppressor gene in gliomas.

Our study demonstrates that ITPKA is markedly downregulated in glioma tissues and that its expression level is strongly correlated with several key clinicopathological indicators, including pathological grade, glioma subtype, and molecular characteristics. To confirm the reliability of the aforementioned conclusions, in addition to analyzing the CGGA database, this study also incorporated the TCGA database for double verification analysis. In comparison with the analytical results from the CGGA database, the TCGA database exhibited the following specific expression features: at the pathological grade level, there was no statistically significant difference in ITPKA expression between WHO grade IV and WHO grade III gliomas (**Supplementary Figure 2A**). Among pathological subtypes, the ITPKA expression level in oligodendroglioma was significantly higher than that in oligoastrocytoma, astrocytoma, and GBM (**Supplementary Figure 2B**). Among molecular subtypes, the ITPKA expression in neuronal glioma was not only higher than that in classical and mesenchymal subtypes but also remarkably elevated compared with the proneuronal subtype (**Supplementary Figure 2C**). In gender stratification analysis, female glioma patients showed a significantly higher ITPKA expression level than male patients (**Supplementary Figure 2E**). Although there were partial discrepancies in the analytical results between the two databases, this inconsistency may be ascribed to multiple factors such as differences in sample sources, sample sizes, and inclusion criteria, which in turn result in divergent analytical outcomes. Notably, the analysis result of the CGGA database further revealed that ITPKA expression in recurrent gliomas was higher than that in primary gliomas, a phenomenon that might be the consequence of adaptive subclones selected by treatment, meaning that during tumor recurrence, the subpopulation of cells with high ITPKA expression survived and proliferated, indirectly confirming the regulatory role of ITPKA in glioma cells. Survival analysis results further confirmed the clinical value of ITPKA: a high ITPKA expression exerted a survival-protective effect in both primary and recurrent gliomas, whereas no correlation between ITPKA expression and survival was observed in secondary gliomas, presumably because of the small sample size of this subtype included in the study. It should also be emphasized that recurrent gliomas are more malignant, which explains why their median OS time was significantly shorter than that of primary gliomas (**Supplementary Figure 3**). Subsequently, our analysis via IHC staining showed that Ki-67, a classic cell proliferation marker, had a stronger expression level in HGGs. Furthermore, overexpression of Ki-67 was significantly associated with poorer OS and PFS in glioma patients, suggesting that it could serve as a potential indicator for glioma prognosis assessment (39); the inverse expression trend between ITPKA and Ki-67 further implies that ITPKA may be involved in the malignant progression of gliomas by inhibiting tumor cell proliferation. From the perspective of clinical treatment, for patients with oligodendroglioma harboring 1p/19q co-deletion, the addition of PCV (procarbazine, lomustine, vincristine) to radiochemotherapy can effectively control disease progression and prolong survival time (40, 41). Our study found that ITPKA was overexpressed in patients with a 1p/19q co-deletion, indicating that the expression level of ITPKA may be associated with the radiochemosensitivity of this glioma subtype, and the underlying molecular mechanism is worthy of in-depth exploration. Furthermore, survival analysis confirmed that glioma patients with high ITPKA expression exhibited significantly prolonged OS.

To further elucidate the functional role of ITPKA in gliomas, we focused on GBM and performed a series of *in vitro* and *in vivo* experiments. Our results confirm that ITPKA acts as a novel tumor suppressor gene in GBM. However, at present, the molecular mechanisms underlying the regulation of the malignant behaviors of glioma cells by ITPKA remain poorly understood. Previous studies have demonstrated that ITPKA can promote tumor cell migration through two distinct mechanisms. First, the protein binds to F-actin via its N-terminal actin-binding domain, while its C-terminal monomeric domain inhibits the formation of F-actin bundles and instead facilitates the assembly of thin, dense F-actin networks. This subsequently induces the formation of lamellipodium-like protrusions, alters the pattern of cytoskeletal rearrangement, and ultimately endows tumor cells with a migratory capacity. Second, ITPKA generates inositol-1,3,4,5-tetrakisphosphate (Ins (1, 3–5)P4) via its catalytic domain and inhibits the activity of inositol 5-phosphatase activity. This process promotes the release of Ca²^+^ from intracellular calcium stores and efficiently activates Ca²^+^ influx, thereby enhancing the migratory capacity of tumor cells (14, 36–38). These properties render ITPKA a potential therapeutic target for tumor invasion and metastasis. The findings of these aforementioned studies further support the *in vitro* results of the present study, indicating that ITPKA can significantly inhibit the proliferative, migratory, and invasive abilities of GBM cells during the malignant progression of GBM. However, the present study did not investigate whether ITPKA mediates the regulation of GBM cell proliferation, migration, and invasion through these two cellular processes by facilitating the assembly of F-actin network assembly and Ca²^+^ influx, and it is highly probable that these same mechanisms also exert regulatory effects in the experimental systems used in this study.

Cell cycle progression and apoptosis play fundamental roles in tumor cell proliferation and tumor progression. The G1/S phase is characterized by the biosynthesis of RNA and proteins as its core feature. Driving the synthesis of structural and enzymatic proteins lays the foundation for the accurate replication of DNA and synchronous synthesis of chromosome-associated proteins (including histones and non-histones). Ultimately, this ensures precise transmission of genetic information during cell division and maintains the stability of heritable traits. In contrast, the core function of the G2 phase is to monitor DNA molecules for damage or lesions, allowing cells with intact DNA to enter the mitotic phase (42, 43). Our study found that ITPKA inhibits GBM cell division by arresting cells in the G1 phase, thereby inducing GBM cell apoptosis *in vitro*. This also provides mechanistic support for the ITPKA-mediated inhibitory effect on GBM cell proliferation. Our *in vivo* experiments confirmed that high ITPKA expression suppressed GBM growth. This result was highly consistent with our *in vitro* observations. In summary, ITPKA inhibits the malignant biological behaviors of GBM through multiple pathways, indicating its clinical potential as a therapeutic target.

Although this study confirms that ITPKA plays a crucial biological role in GBM, our research has unavoidable limitations that warrant further in-depth investigations. First, the retrospective analysis in this study relied on existing clinicopathological data. Public database platforms contain numerous uncontrollable confounding factors, such as incomplete clinical characteristics of patients and the presence of other therapeutic interventions, which may introduce bias into the study results. Second, previous studies have shown that ITPKA is regulated by two transcription factors (p53 and GATA3) and is enriched in eight glioma-related subpathways within the inositol phosphate metabolism pathway (21). Thus, the regulatory pattern of ITPKA in these relevant subpathways, and its interaction with other genes and signaling pathways should further be verified. Third, bioinformatic analysis reveals that ITPKA is correlated with the degree of infiltration of certain immune cells into the tumor microenvironment. For instance, ITPKA was positively correlated with the degree of infiltration of CD4+ and CD8+ T cells, and negatively correlated with the degree of infiltration of dendritic cells (**Supplementary Figure 4**). Based on this, it is hypothesized that ITPKA may be involved in the tumor immune regulation process and influence the regulatory mechanism of immune cell infiltration in gliomas. However, the present study only discussed the role of ITPKA in the migration and invasion of glioma cells, and the aforementioned immune regulation-related mechanisms must be further explored in subsequent studies. Finally, this study draws conclusions regarding the regulatory role of the ITPKA gene in the tumorigenic ability and tumor growth of GBM using a subcutaneous xenograft tumor model in nude mice. The present study verified that ITPKA exerted a regulatory effect on GBM growth in this tumor model. However, it should be noted that although subcutaneous xenograft GBM model in nude mice has used in some recent studies (22, 25, 27), the tumor microenvironment of subcutaneous xenograft differs significantly from the intracranial environment of the primary glioma in clinical settings. The glioma microenvironment is characterized by highly complex biological features including extensive tumor heterogeneity, specific immunosuppressive cellular components, unique regulatory functions of the blood–brain barrier, close associations between molecular classification and the immune microenvironment, and dual systemic and local immunosuppression (44). Therefore, whether the conclusions drawn from this subcutaneous xenograft model truly reflect the pathological characteristics and disease progression patterns of patients with clinical gliomas warrants further validation in future studies.

## Data Availability

The original contributions presented in the study are included in the article/**Supplementary Material.** Further inquiries can be directed to the corresponding author/s.
