# Supplementary material for: ITPKA suppresses glioma progression and predicts patient prognosis
Source: Front Oncol. 2026 Apr 22;16:1802857. doi: 10.3389/fonc.2026.1802857 (PMC13143765; doi:10.3389/fonc.2026.1802857)
Supplement: Supplementary file 1 [file Supplementaryfile1.docx]

Supplementary Material

# Supplementary Figures


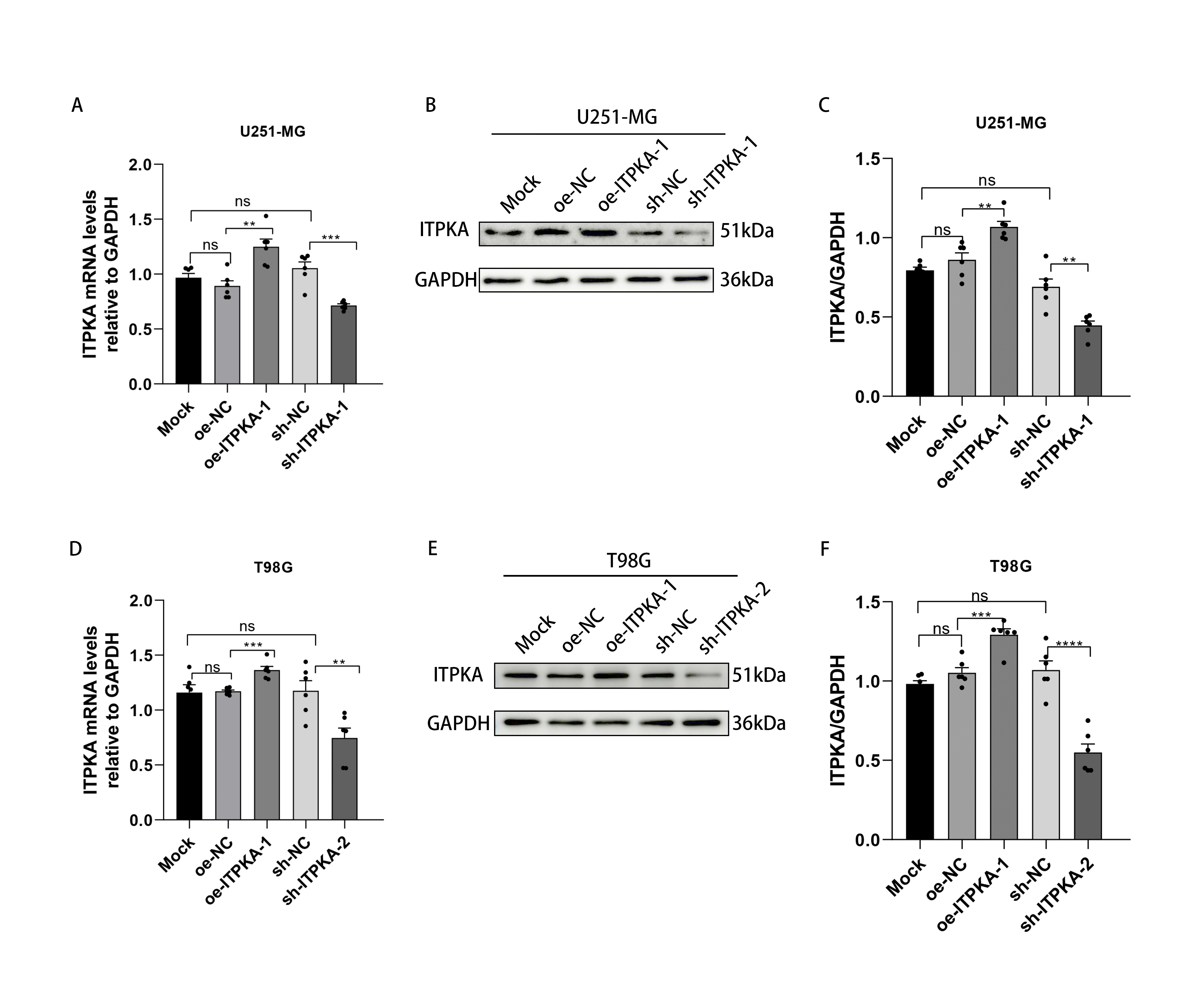


**Supplementary Figure 1.** **Verification of ITPKA protein expression in U251-MG and T98G cell lines after transfection manipulation.** (**A**-**C**) RT-qPCR (A) or Western blotting (B-C) shows the relative expression difference of ITPKA in U251-MG cells under Mock, oe-NC, oe-ITPKA-1, sh-NC and sh-ITPKA-1 conditions. (**D**-**F**) RT-qPCR (D) or Western blotting (E-F) shows the relative expression difference of ITPKA in T98G cells under Mock, oe-NC, oe-ITPKA-1, sh-NC and sh-ITPKA-2 conditions. Data presented as mean ± SD; the Student’s t-test and one-way analysis of variance (ANOVA) followed by multiple comparison tests were conducted. The experiments were independently repeated at least three times. ns: not significant; **p < 0.01, ***p < 0.001, ****p < 0.0001.

**
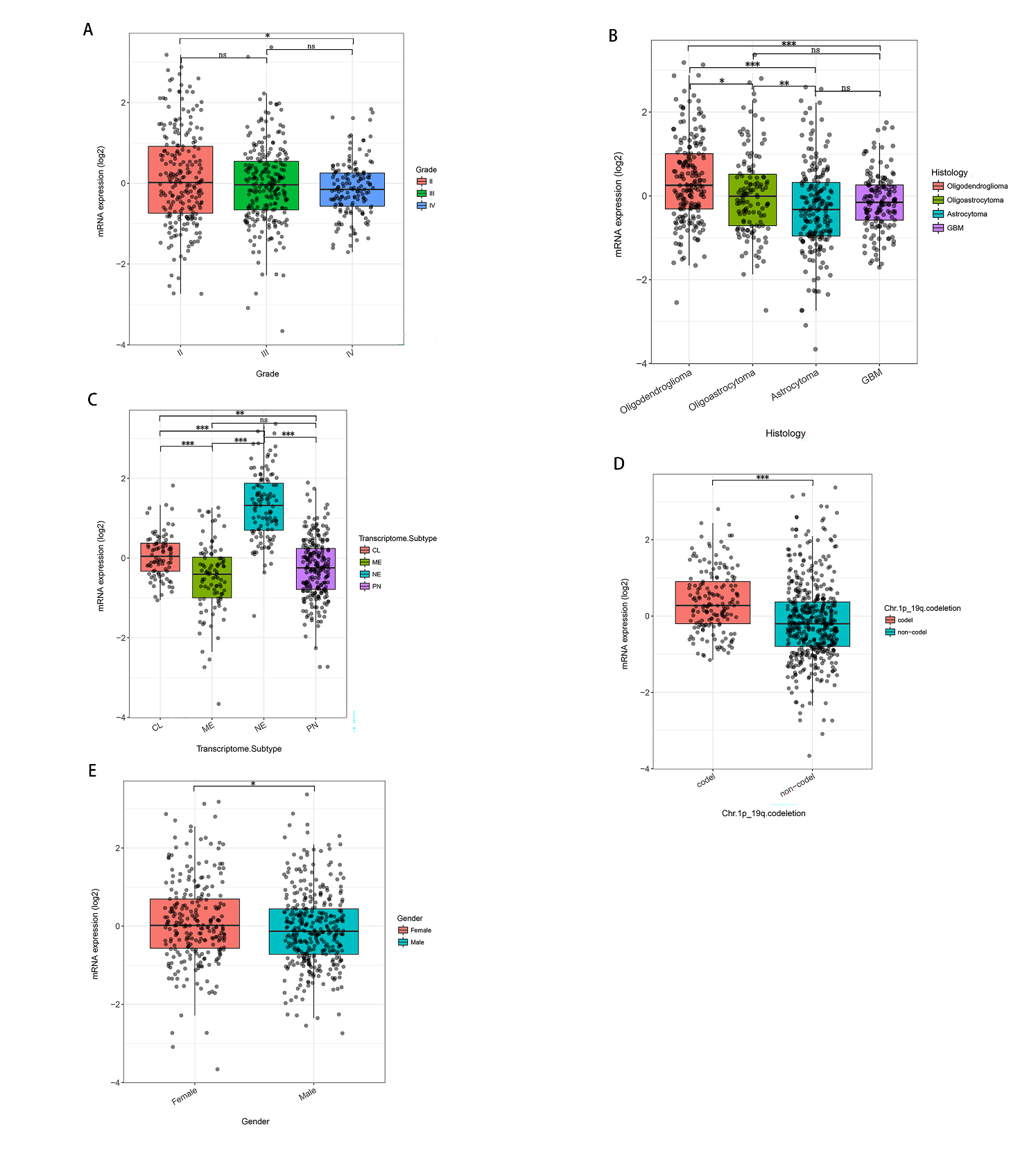
**

**Supplementary Figure 2.** **The expression level of ITPKA gene in glioma tissues within The Cancer Genome Atlas (TCGA) database is closely correlated with the clinicopathological characteristics of patients.** (**A**) shows the relative expression differences of ITPKA gene mRNA in glioma patients with different World Health Organization (WHO) grades Ⅱ, Ⅲ, and Ⅳ in the TCGA database. (**B**) shows the relative expression differences of ITPKA gene mRNA in glioma patients with different histological types (oligodendroglioma, oligoastrocytoma, astrocytoma, and glioblastoma) in the TCGA database. (**C**) shows the relative expression differences of ITPKA gene mRNA in glioma patients with different transcriptome subtypes (CL, ME, NE, and PN subtypes) in the TCGA database. (**D**) shows the relative expression differences of ITPKA gene mRNA in glioma patients with 1p/19q chromosome codeletion and non-codeletion in the TCGA database. (**E**) shows the relative expression differences of ITPKA gene mRNA in glioma patients of different genders (female and male) in the TCGA database. Data presented as mean ± SD; ns: not significant; *p < 0.05, **p < 0.01, ***p < 0.001. CL: Classic; ME: Mesenchymal; NE: Neural; PN: Proneural

**
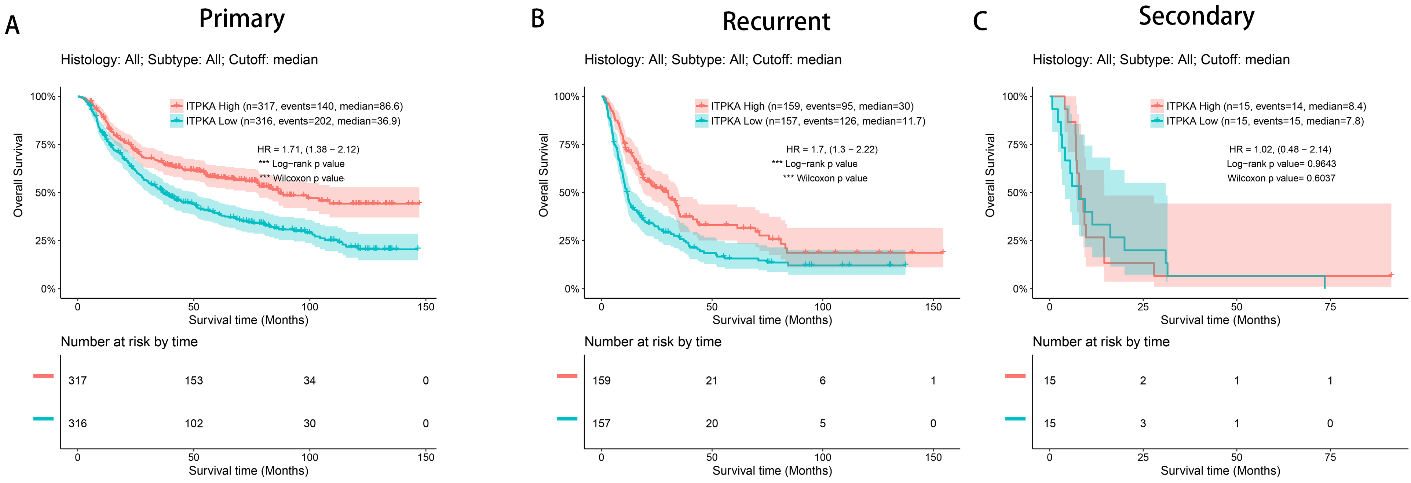
Supplementary Figure 3.** **The association between ITPKA gene expression level and overall survival in patients with different subtypes of glioma in the Chinese Glioma Genome Atlas (CGGA) database.** (**A**) shows the survival analysis curve of ITPKA expression in patients with primary glioma. (**B**) shows the survival analysis curve of ITPKA expression in patients with recurrent glioma. (**C**) shows the survival analysis curve of ITPKA expression in patients with secondary glioma. Data presented as mean ± SD; ns: not significant; ***p < 0.001.


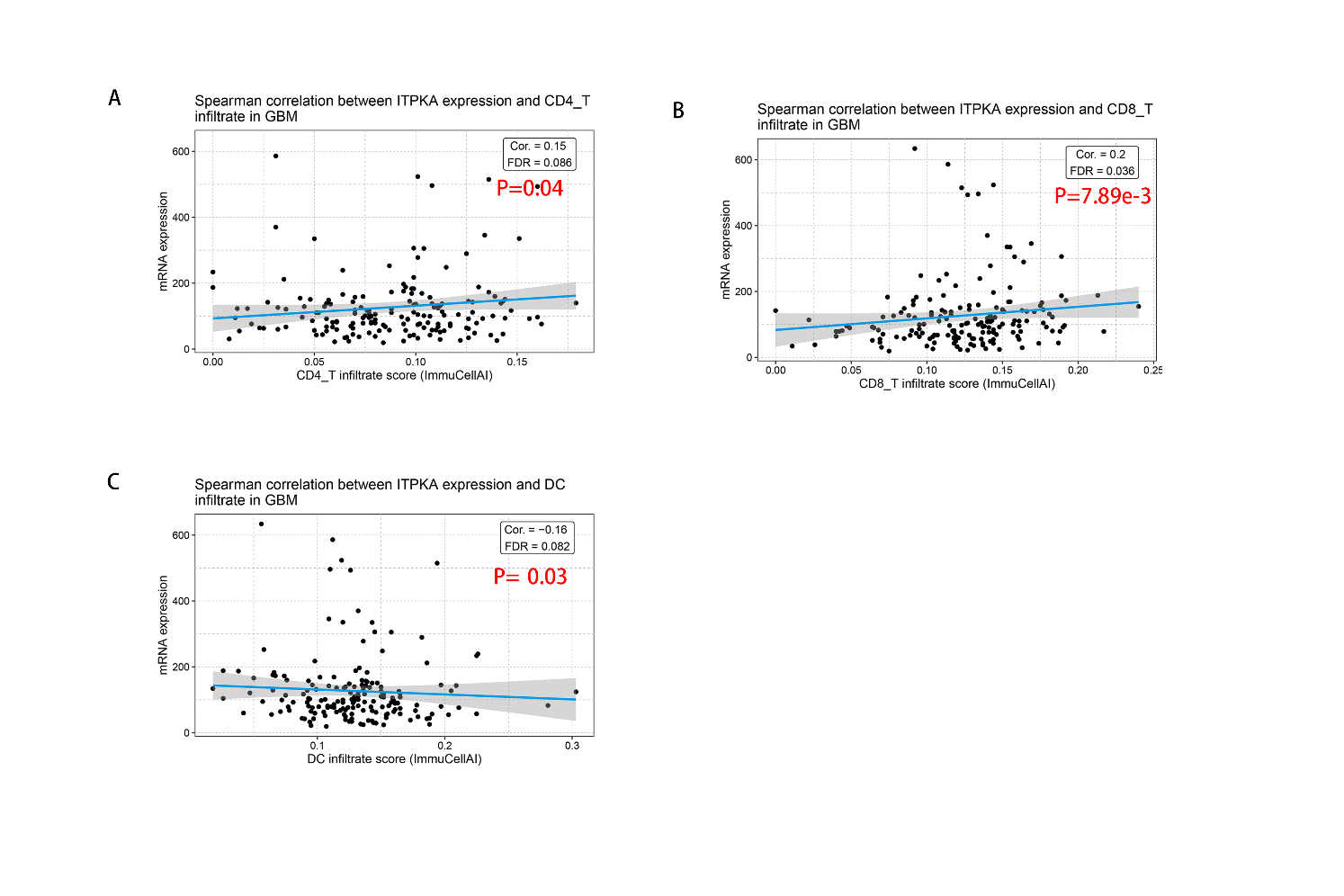


**Supplementary Figure 4. The Spearman correlation analysis for the association between ITPKA gene mRNA expression level and immune cell infiltration level in the tissues of glioblastoma (GBM) patients in The Cancer Genome Atlas (TCGA) database.** (**A**) shows the Spearman correlation between ITPKA mRNA expression and CD4⁺ T cell infiltration score in GBM. (**B**) shows the Spearman correlation between ITPKA mRNA expression and CD8⁺ T cell infiltration score in GBM. (**C**) shows the Spearman correlation between ITPKA mRNA expression and dendritic cell (DC) infiltration score in GBM.
