# Supplementary material for: ITPKA suppresses glioma progression and predicts patient prognosis
Source: Front Oncol. 2026 Apr 22;16:1802857. doi: 10.3389/fonc.2026.1802857 (PMC13143765; doi:10.3389/fonc.2026.1802857)
Supplement: Supplementary file 2 [file Table1.docx]

Supplementary Material

# Supplementary Table


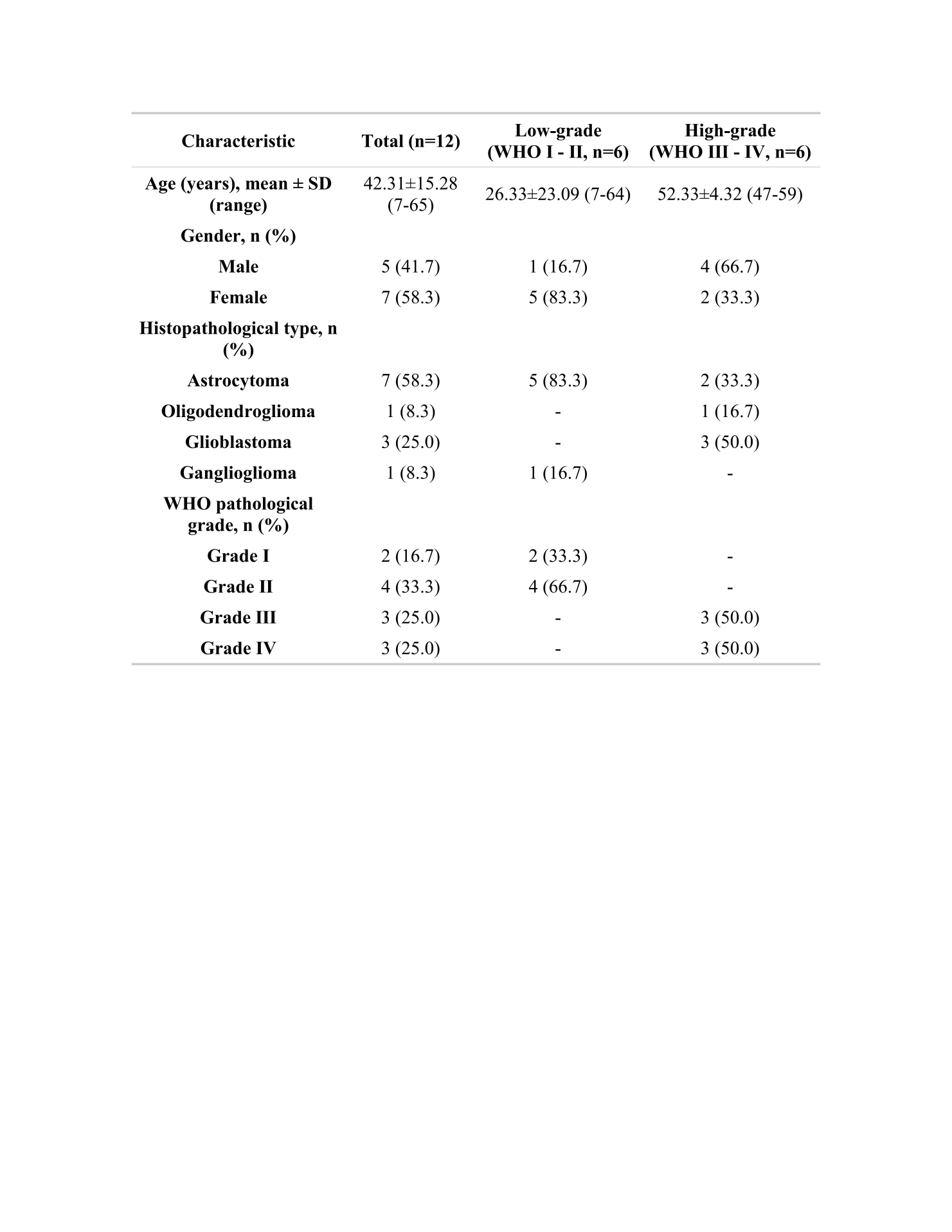


**Supplementary Table 1.** **Clinicopathological characteristics of glioma patients stratified by World Health Organization (WHO) grade (n=12).** Continuous variables (age) are expressed as mean ± standard deviation (SD) with ranges in parentheses, and categorical variables as n (%). Low-grade gliomas were defined as WHO grade Ⅰ – Ⅱ, and high-grade gliomas as WHO grade Ⅲ – Ⅳ. “-” denotes no available data.
